# *corona* Is Required for Higher-Order Assembly of Transverse Filaments into Full-Length Synaptonemal Complex in Drosophila Oocytes

**DOI:** 10.1371/journal.pgen.1000194

**Published:** 2008-09-19

**Authors:** Scott L. Page, Radhika S. Khetani, Cathleen M. Lake, Rachel J. Nielsen, Jennifer K. Jeffress, William D. Warren, Sharon E. Bickel, R. Scott Hawley

**Affiliations:** 1 Comparative Genomics Centre, School of Pharmacy and Molecular Sciences, James Cook University, Townsville, Australia; 2 Stowers Institute for Medical Research, Kansas City, Missouri, United States of America; 3 Department of Biological Sciences, Dartmouth College, Hanover, New Hampshire, United States of America; 4 Department of Physiology, Kansas University Medical Center, Kansas City, Kansas, United States of America; The University of North Carolina at Chapel Hill, United States of America

## Abstract

The synaptonemal complex (SC) is an intricate structure that forms between homologous chromosomes early during the meiotic prophase, where it mediates homolog pairing interactions and promotes the formation of genetic exchanges. In *Drosophila melanogaster*, C(3)G protein forms the transverse filaments (TFs) of the SC. The N termini of C(3)G homodimers localize to the Central Element (CE) of the SC, while the C-termini of C(3)G connect the TFs to the chromosomes via associations with the axial elements/lateral elements (AEs/LEs) of the SC. Here, we show that the Drosophila protein Corona (CONA) co-localizes with C(3)G in a mutually dependent fashion and is required for the polymerization of C(3)G into mature thread-like structures, in the context both of paired homologous chromosomes and of C(3)G polycomplexes that lack AEs/LEs. Although AEs assemble in *cona* oocytes, they exhibit defects that are characteristic of *c(3)G* mutant oocytes, including failure of AE alignment and synapsis. These results demonstrate that CONA, which does not contain a coiled coil domain, is required for the stable ‘zippering’ of TFs to form the central region of the Drosophila SC. We speculate that CONA's role in SC formation may be similar to that of the mammalian CE proteins SYCE2 and TEX12. However, the observation that AE alignment and pairing occurs in *Tex12* and *Syce2* mutant meiocytes but not in *cona* oocytes suggests that the SC plays a more critical role in the stable association of homologs in Drosophila than it does in mammalian cells.

## Author Summary

Meiosis is a specialized type of cell division that is needed to produce sperm and egg cells, which carry only half the number of chromosomes of other cells in the body. Meiosis is required for reproduction, but abnormalities in chromosome number caused by errors in the process of meiosis are responsible for many birth defects and mental retardation syndromes in humans. The fruit fly, *Drosophila melanogaster*, is an excellent organism in which to study meiosis because of the powerful genetic and microscopic techniques that can be implemented with it. Early in meiosis, homologous chromosomes are joined together by an elaborate protein structure called the synaptonemal complex (SC) that plays a critical role in both holding homologous chromosomes together and in facilitating a process known as meiotic recombination. In this study, we have found a protein called Corona that is required for the formation of the SC. Our data show that Corona is required for the proper localization of the SC protein C(3)G. In the absence of Corona, C(3)G fails to polymerize and form the central region of the SC. Increasing our understanding of SC assembly and function will lead to a better understanding of the mechanism for proper chromosome segregation during meiosis.

## Introduction

During meiosis, the diploid genome is segregated to form haploid nuclei through processes that include the close juxtaposition of homologous chromosomes and recombination between them. In most organisms, a proteinaceous structure called the synaptonemal complex (SC) forms between homologous chromosomes during meiotic prophase. The SC is required for synapsis, the intimate association of homologs along their entire length. The SC and its components are thought to play roles in regulating recombination and generally promoting the establishment of crossovers between the chromosomes [1],[2].

Examination of SCs by electron microscopy (EM) has defined distinct structures present in the SCs of most organisms. During early prophase, axial elements (AEs) form along the longitudinal axis of each pair of sister chromatids using a cohesin-based chromosome core as a scaffold for assembly [3]. As prophase progresses, the AEs of homologous chromosomes become physically connected by perpendicular transverse filaments (TFs) that span the SC central region (CR), which occupies the ∼100 nm space between the two homologous AEs [1],[2]. AEs within the mature SC are referred to as lateral elements (LEs). Finally, a central element (CE) is often observed as a structure overlapping the middle of the TFs and positioned parallel to the two LEs.

Although homologous chromosomes undergo presynaptic pairing and alignment in some organisms [4],[5], synapsis requires a fully formed CR that extends the length of the chromosomes. In this paper we will use the term “alignment” to describe the parallel association of homologs (or AEs) at a distance equal to or greater than the width of the SC and the term “pairing” to describe the close association of homologous sequences as determined by FISH.

Components of TFs, such as ZIP1 (budding yeast), SYCP1 (mouse), SYP-1 (worms), and C(3)G (Drosophila), have been identified as proteins containing long coiled coil segments [6]–[11]. Although these TF proteins are similar in predicted secondary structure, they share very little similarity in amino acid sequence. Despite this lack of sequence conservation, the proteins are all thought to form TFs across the CR of the SC by binding of their C-termini to the AEs with head-to-head orientation of their N-termini at the center of the CE [12]–[16]. TFs are important for ensuring synapsis of homologs and normal levels of interhomolog exchange [6], [8], [10], [17]–[20].

In *Drosophila melanogaster* oocytes, the TFs are formed by the C(3)G protein [8],[12]. Like other TF proteins, C(3)G is comprised of a central coiled coil-rich domain flanked by N- and C-terminal globular domains. As shown by Jeffress *et al.* [21], C-terminal deletion of C(3)G results in its failure to attach to the AEs of each set of homologs. Instead, this C-terminal deletion protein forms a large cylindrical polycomplex structure. EM analysis of this structure reveals a polycomplex of concentric rings with alternating dark and light bands, presumably corresponding to long arrays of polymerized TFs. However, deletions of N-terminal regions completely abolished both SC and polycomplex formation. To explain these data, Jeffress *et al.* [21] proposed that in Drosophila, the N- terminal globular domain of C(3)G is critical for the formation of anti-parallel pairs of C(3)G homodimers, and thus for assembly of complete TFs, while the C-terminus is required to affix these homodimers to the AEs.

The question then arises as to how C(3)G molecules can be polymerized to form a linear array of TFs. The idea that such polymerization events are facilitated by the apposition of paired AEs seems unlikely given the finding that C-terminal deletions of C(3)G form polycomplexes [21]. The observation that the rat homolog of C(3)G (SYCP1) can form polycomplex-like structures in COS-7 cells [22] suggests that the process of TF polymerization may be self-promoting and sustaining, and thus requires no other components. However, in mice, significant extension of SYCP1 to form a full-length CR in meiotic cells requires the functions of the SYCE1, SYCE2, and TEX12 proteins, which localize to the CE of the SC [23]–[26]. SYCE1 and SYCE2 physically associate with each other and the N-terminus of the TF protein SYCP1, while TEX12 binds to SYCE2 [24],[25]. Mice lacking the SYCE2 protein display defects in the formation of the TFs (SYCP1 accumulation), and thus in SC formation [23]. They appear to form only short and, at least in the case of *Tex12^−/−^* mice, morphologically abnormal SCs [23],[26]. It remains to be determined whether or not functional homologs of SYCE2 and TEX12 might facilitate C(3)G polymerization, and thus CE formation in Drosophila oocytes.

To discover additional components of the SC and genes involved in other critical processes in meiosis, we previously undertook a novel genetic screen for female meiotic mutants in Drosophila [27]. One of the genes identified in the screen, *corona* (*cona*), was found to have both a severe defect in meiotic recombination and a profound effect on the localization of C(3)G. Previous analyses of *cona* mutants demonstrated a failure of the SC protein C(3)G to localize correctly in the absence of CONA, demonstrating defective SC formation. As is the case for *c(3)G* mutants, the frequency of meiotic exchange in *cona* females was reduced 50- to 200-fold compared to wild-type [27] without a similar reduction in the number of DSBs [SLP and RSH, unpublished data]. Moreover, double mutants for *c(3)G* and *cona* displayed a defect in recombination that was comparable to either single mutant [SLP and RSH, unpublished data], and thus the two proteins likely function in a common pathway with respect to facilitating meiotic exchange. Like C(3)G, CONA protein is only conserved within the genus Drosophila, but CONA contains no predicted coiled coil domains or other characterized functional motifs [27].

In this study, we show that CONA is a new SC protein that co-localizes with C(3)G in a mutually-dependent fashion. We found that CONA accumulation is required for C(3)G localization into wild-type SC structures and formation of polycomplexes, but is not necessary for the formation of either the AEs or the chromosome cores from which they arise. Our results indicate that CONA is crucial for the assembly of the CR of the SC in Drosophila and may have a function similar to that of the vertebrate CE proteins TEX12 and SYCE2. However, the observation that pairing and alignment of AEs occurs in *Tex12* and *Syce2* mutant meiocytes, but not in *cona* oocytes, suggests that the SC plays a more critical role in the stable association of homologs in Drosophila than it does in mammalian cells.

## Results

### Corona Protein Co-Localizes with the Synaptonemal Complex

We previously showed that the *cona* gene corresponds to the transcription unit *CG7676* on the basis of the presence of a *Doc* transposon in the 3′ untranslated region of *CG7676* in *cona^A12^* that was not present on the un-mutagenized parental chromosome and the isolation of a second allele, *cona^f04903^*, which bears a *PiggyBac* insertion in sequence flanking the 5′ end of *CG7676* [27]. Both *cona^A12^* and *cona^f04903^* drastically reduce the levels of meiotic recombination and produce high levels of nondisjunction (∼32–39%) [27, SLP and RSH, unpublished data].

**Figure 1:**
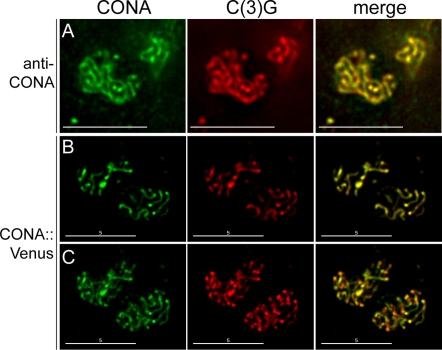
CONA protein co-localizes with C(3)G. (A) Wild-type pro-oocytes stained with anti-CONA and anti-C(3)G, showing CONA (green) and C(3)G (red) co-localization. (B) Images of a single deconvolved optical section of a pair of pro-oocytes showing that CONA::Venus (green) and C(3)G (red) co-localize extensively. (C) Maximum intensity projections of the entire nuclei from B. Scale bars, 5 µm.

We raised an antibody against the CONA protein and used it to determine the localization of CONA in meiotic prophase cells in the germaria of Drosophila ovaries (see Materials and Methods). Evidence that this antibody is specific to CONA (i.e., that no signal is observed in pro-oocytes homozygous for *cona^f04903^*) is presented in Figure S1. In wild-type ovaries, anti-CONA localization was observed within a subset of nuclei in regions 2A and 2B of the germarium and within the oocyte nucleus in region 3 and early egg chambers within the vitellarium. The distribution of CONA within nuclei was distinctly thread-like and strongly co-localized with the SC protein C(3)G (Figure 1A). These results demonstrate that CONA localizes along the SC.

As an alternate strategy to localize the protein, we constructed a transgene, *P{UASP-cona::Venus}*, which expresses the full-length CONA protein fused to the yellow fluorescent protein Venus under the control of the GAL4/UAS system [28]. The CONA::Venus fusion protein was functional, as expression driven by *nanos*-GAL4::VP16 in the female germline rescued the nondisjunction phenotype in *cona^f04903^* homozygotes. Control *cona^f04903^* homozygotes lacking the *P{UASP-cona::Venus}* transgene showed 31.9% *X* chromosome nondisjunction, whereas *cona^f04903^* homozygotes expressing CONA::Venus showed a nearly tenfold reduction in nondisjunction to just 3.4% (data not shown).

We examined the pattern of *nanos*-GAL4::VP16-driven CONA::Venus localization during meiotic prophase. In a *cona^f04903^* mutant background, strong Venus yellow fluorescent protein signal localized in a pattern very similar to that observed for CONA immunolocalization. Immunolocalization of C(3)G in these ovaries revealed extensive co-localization of CONA::Venus and C(3)G in thread-like patterns within nuclei (Figure 1B–C). Nuclear CONA::Venus fluorescence was strongest in a *cona* mutant background in which little or no wild-type CONA protein is present (Figure S1). When expressed in heterozygotes or homozygotes for a wild-type copy of the *cona* locus, CONA::Venus fluorescence was weaker in nuclei and increased diffuse fluorescence was often observed in the cytoplasm of germline cells in regions 1 and 2 of the germarium (data not shown). This may be the result of competition with wild-type CONA protein. A similar reduction in signal has been observed for localization of GFP-tagged ORD protein along the SC in the presence of wild-type ORD (RSK and SEB, unpublished data). These data confirm the immunolocalization of CONA and implicate CONA as a component of the SC.

### CONA Is Required for the Assembly of C(3)G into a Thread-Like SC

**Figure 2:**
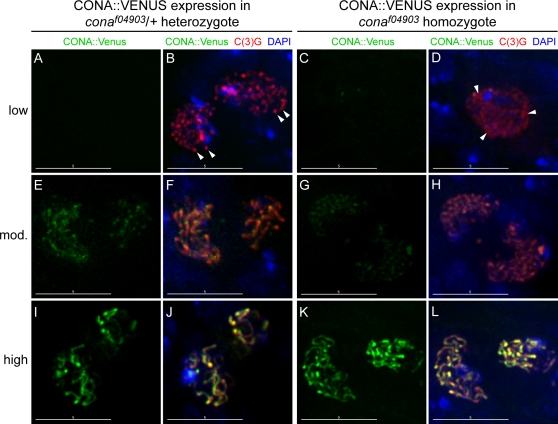
CONA is required for the thread-like localization of C(3)G. Shown is the localization of CONA::Venus (green), C(3)G (red), and DAPI (blue) in region 2A of germaria from *P{nos-GAL4::VP16}/+*; *P{UASP-cona::Venus}/+* ; *cona^f04903^/+* (A, B, E, F, I, J, left) and *P{nos-GAL4::VP16}/+* ; *P{UASP-cona::Venus}/+* ; *cona^f04903^* (C, D, G, H, K, L, right). The top, middle and bottom rows show pro-oocytes in which C(3)G was present but CONA::Venus was visible at very low to undetectable (A, B, C, D), moderate (mod.) (E, F, G, H), or high (I, J, K, L) levels. When one functional copy of the endogenous *cona^+^* gene is present, the localization of C(3)G takes on a punctate to thread-like pattern (arrowheads) in very early cysts, even when CONA::Venus is not readily detected (A, B). The spotty to thread-like pattern of C(3)G accumulation observed in panel B is also observed in early region 2A in *cona^f04903^/+* heterozygotes that lack the CONA::Venus construct, and represents an early stage (zygotene) in SC assembly (Figure S1C). When CONA::Venus is expressed in a *cona* homozygous mutant background and is the only functional CONA protein present, the initial localization of C(3)G resembles that of a *cona* mutant homozygote (C, D), with diffuse and spotty regions of C(3)G localization (arrowheads). C(3)G takes on a thread-like appearance only when CONA::Venus begins to be detected (G, H, K, L), showing that the assembly of C(3)G into a thread-like SC coincides with and requires the accumulation of CONA::Venus protein. Scale bars, 5 µm.

When CONA::Venus was expressed under the control of a *nanos*-GAL4::VP16 driver in a *cona^f04903^* heterozygote in which wild-type CONA protein was also present, C(3)G was detected as puncta and short threads within early prophase nuclei before CONA::Venus signal was detectable (Figure 2A–B). The spotty to thread-like pattern of C(3)G accumulation observed in Figure 2B is also observed in early region 2A in *cona^f04903^/+* heterozygotes that lack the CONA::Venus transgene, and represents an early stage (zygotene) in SC assembly in which the short threads of C(3)G are associated with endogenous CONA (Figure S1C). As the intensity of CONA::Venus staining increased during the progression of meiotic prophase, CONA::Venus assumed a thread-like staining pattern that co-localized with C(3)G (Figure 2E–F, 2I–J).

A different pattern of C(3)G localization was observed when CONA::Venus was expressed in the *cona^f04903^* homozygote, and therefore was the only form of functional CONA protein present. In nuclei that contained very low or undetectable levels of CONA::Venus signal (Figure 2C–D), C(3)G staining exhibited a more diffuse appearance similar to that previously described for *cona* mutant pro-oocytes [27]. However, as CONA::Venus staining became more visible at slightly later stages, CONA::Venus and C(3)G co-localized in short thread-like segments and the diffuse C(3)G signal was no longer observed (Figure 2G-H). Eventually, CONA::Venus and C(3)G co-localization resembled that observed in the *cona^f04903^* heterozygote in pachytene nuclei with fully assembled SC (Figure 2K–L). These data further demonstrate that the assembly of C(3)G into a thread-like SC requires the accumulation of CONA and involves co-localization with the CONA protein.

### Corona Localization Mimics that of C(3)G when AE Components Are Mutated

The AEs are believed to form from chromosome core structures that contain sister chromatid cohesion proteins [3]. In mammals, AE-specific proteins such as SYCP2 and SYCP3 associate with components of the cohesin complex during the initial steps of SC assembly [13], [29]–[37]. Similarly, cohesin-based chromosomal cores/AEs form in Drosophila pro-oocytes [38]. Formation of the chromosomal core in Drosophila is dependent on the product of the *c(2)M* gene, which also localizes along this structure [12],[38],[39]. ORD protein also localizes along chromosome cores and is required for the maintenance of chromosome core integrity during meiotic prophase [38],[40]. Mutants in AE/LE proteins often result in recombination defects and the failure of synapsis, which indicates that properly formed AEs/LEs are required for the normal formation of the SC central region [31], [39], [41]–[44].

To better understand the association of CONA with the SC, we examined the localization of the CONA protein in mutants that disrupt different components of the AE. Mutations in the *c(2)M* gene result in incomplete SC formation, as indicated by only very short segments of nuclear C(3)G localization, in contrast to the long, thread-like localization observed in wild-type [39]. In contrast, in *ord* mutants, the thread-like C(3)G staining appears to disassemble earlier than in wild-type due to the dissolution of cohesin-based chromosome cores along the chromosome arms [38],[40].

**Figure 3:**
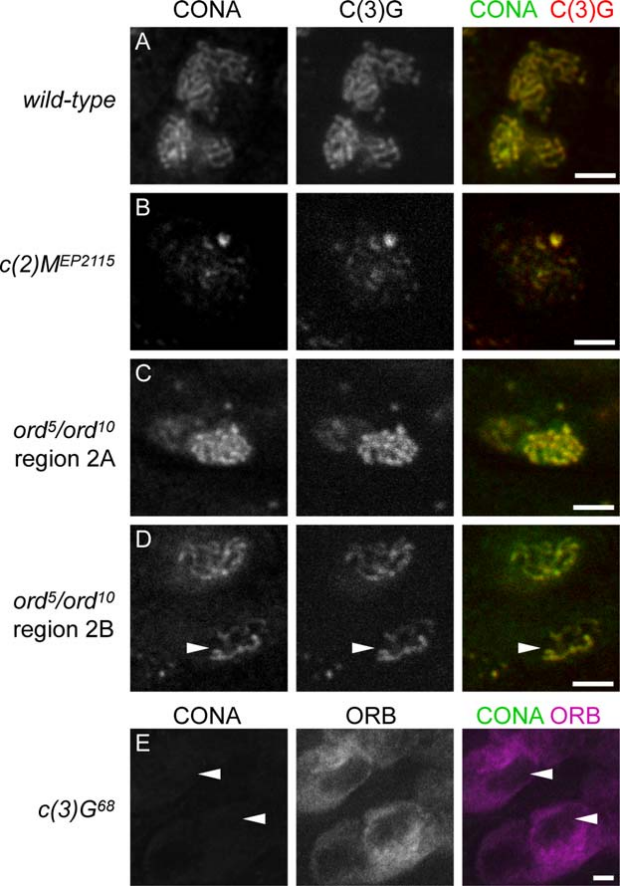
CONA co-localizes with C(3)G in disrupted SCs and requires C(3)G for localization. (A) Wild-type control pro-oocytes stained with anti-CONA and anti-C(3)G, showing CONA and C(3)G co-localization. (B) *c(2)M^EP2115^* homozygous pro-oocyte stained to detect CONA and C(3)G co-localization. (C) *ord^5^/ord^10^* pro-oocytes from germarium region 2A showing CONA and C(3)G co-localization. (D) *ord^5^/ord^10^* pro-oocytes from germarium region 2B (anterior tip of germarium oriented toward the top) stained to detect CONA and C(3)G co-localization in pro-oocytes experiencing early SC disassembly (arrowhead). (E) *c(3)G^68^* homozygote germarium showing the absence of CONA signal in pro-oocytes (arrowheads) marked by high levels of cytoplasmic ORB protein. Scale bars, 5 µm.

Analysis of CONA localization in *c(2)M^EP2115^* homozygous pro-oocytes demonstrated that CONA was localized in numerous short segments that corresponded to sites of C(3)G localization (Figure 3A). CONA was consistently co-localized with C(3)G and was not seen to localize elsewhere in the germarium except to the dot-like short segments of C(3)G. The observed localization of CONA in *c(2)M^EP2115^* homozygotes could indicate a robust association with C(3)G and/or an inability to localize to abnormally formed AEs except at sites stabilized by C(3)G accumulation. Nonetheless, the dependency on AEs for localization is similar for both CONA and C(3)G.

CONA localization was also analyzed in *ord^5^/ord^10^* trans-heterozygotes, in which no *ord* activity is present [40]. In agreement with published data [40], we found that C(3)G formed extensive thread-like patterns of localization in pro-oocyte nuclei in region 2A (Figure 3B), but appeared as shorter segments in older germline cysts beginning in late region 2B (Figure 3C). Oocyte nuclei in region 3 displayed C(3)G signals that were further shortened or dot-like, indicating early SC disassembly. At all stages, CONA was always observed to co-localize with C(3)G within the germarium. The initial co-localization of CONA with C(3)G in region 2A was thread-like, similar to wild-type, but older germline cysts did not reveal differences in the extent of localization of the two proteins, suggesting that removal of CONA occurred contemporaneously with C(3)G removal. These results indicate that CONA and C(3)G behave similarly in both *c(2)M* and *ord* mutant backgrounds and suggest that CONA and C(3)G may comprise parts of the same SC sub-structure.

### CONA Requires C(3)G for Localization to the SC

The consistent co-localization of CONA and C(3)G in wild-type and mutant backgrounds and the requirement of CONA for proper C(3)G localization prompted the question of whether C(3)G is required for CONA localization. If CONA is a protein of the AE/LE that is required for C(3)G attachment, it would be expected to localize to chromosomes regardless of whether C(3)G is present or not. When CONA localization was examined in females homozygous for the null mutation *c(3)G^68^*, we found no evidence of CONA antibody staining in pro-oocyte nuclei (Figure 3D). This result is unlike that observed for the AE/LE component C(2)M [21],[39] and suggests that CONA does not act as an AE/LE component that localizes independently of C(3)G. Instead, these data are consistent with a role for CONA within the CR of the SC, which would not be expected to form in the absence of C(3)G.

### SMC1, ORD, and C(2)M Localize to Chromosome Cores in *cona* Mutant Pro-Oocytes

**Figure 4:**
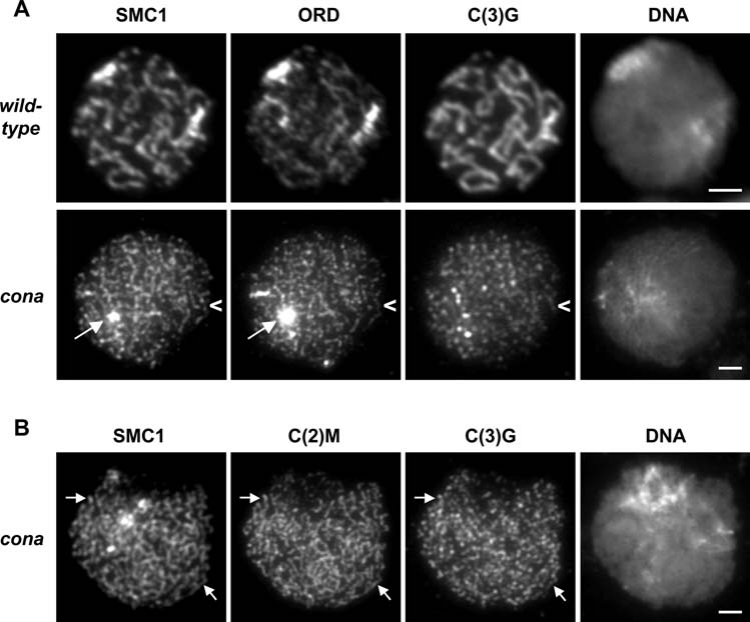
Cohesion and AE proteins localize to chromosomes and form chromosome cores during early prophase in the absence of CONA activity. (A) Localization of SMC1, GFP-ORD, C(3)G and DNA (DAPI) on chromosome spread preparations from wild-type and *cona^A12^/cona^f04903^* mutant ovaries. As in wild-type, SMC1 and GFP-ORD are enriched at centromeres (bright regions, arrows) and localize along the chromosome cores in the *cona* mutant. However, the threads of SMC1 and GFP-ORD localization appear thinner and more numerous than in wild-type, giving them a somewhat disorganized appearance. C(3)G is associated with the chromatin but does not form long thread-like stretches. Although the coincidence of the three proteins is less obvious than in wild-type, short stretches of C(3)G co-localization with the chromosome cores are visible (arrowheads). (B) Localization of SMC1, C(2)M, C(3)G and DNA (DAPI) on chromosome spread preparations of *cona^A12^* ovaries. Like SMC1 and GFP-ORD, C(2)M localizes along the chromosome cores/AEs and co-localization of C(2)M with SMC1 and C(3)G is visible (arrows). The C(3)G signal in *cona* mutants is weaker than in wild-type and has been significantly enhanced to ensure that the details of the staining pattern are visible. The disorganized appearance of cores in both A and B is consistent with absence of AE alignment and synapsis and is similar to that observed for SMC1 and C(2)M localization in *c(3)G* mutant oocytes [38],[39]. All panels are single optical sections. Scale bars, 2 µm.

To further investigate the role of CONA in SC formation, we investigated whether the SC protein C(2)M and the cohesion proteins ORD and SMC1 are able to localize normally in the absence of wild-type *cona*. All three proteins associate with the AEs/LEs of the SC in wild-type [12],[40]. In these experiments, we considered two aspects of ORD, SMC1, and C(2)M localization: first, whether the proteins localized to chromosomes and second, whether the localization appeared equivalent to that observed in wild-type in which normal SC is present. We utilized chromosome spread preparations in which soluble nuclear proteins are removed and only chromosome-associated proteins remain [38]. As shown in Figure 4A, SMC1 and ORD are able to stably associate with the meiotic chromosomes in *cona* mutant pro-oocytes. Both cohesion proteins accumulate normally at the centromeres as evidenced by the bright foci present in both wild-type and *cona* mutant nuclei. Although distinct thread-like staining along the chromosome cores is also visible, the threads of staining appear to be thinner and more numerous than those in wild-type. A similar pattern was also observed for C(2)M localization (Figure 4B). These data suggest that ORD, SMC1, and C(2)M localize to chromosomes and form chromosome cores/AEs in the absence of CONA. However, their localization does not appear equivalent to wild-type, most likely because the AEs do not align and pair. A similar localization pattern for AE/LE proteins has been reported for *c(3)G* mutant oocytes [38],[39].

We also examined C(3)G localization to determine whether C(3)G protein can associate with chromatin in the absence of CONA. Although the C(3)G signal on *cona* spreads is diminished compared to wild-type, and long continuous thread-like staining is absent, puncta and short fragments of chromosome-associated C(3)G are visible. In many cases, these short stretches coincide with C(2)M, SMC1, and ORD (Figure 4A–B). Together, these results argue that CONA is not required for the localization of ORD, SMC1, or C(2)M to chromosomes or for the formation of the AEs. However, our data suggest that in the absence of CONA activity, association of C(3)G with AEs is insufficient for assembly of a normal SC central region and the pairing/alignment of AEs.

### Corona Localizes to C(3)G^Cdel^ Polycomplexes and Is Required for Their Formation

The co-localization with C(3)G, the profound effect on C(3)G localization, and the minor effect on AE protein localization led us to postulate that CONA localizes within the CR of the SC rather than along AEs. Based on this hypothesis, we predicted that CONA would co-localize with C(3)G protein that is prevented from binding to AEs. C(3)G is thought to interact with AEs via its C-terminal globular domain, which is normally oriented toward the AEs [12]. Jeffress and colleagues [21] found that a deletion of amino acids 651–744 at the C-terminal end of C(3)G (known as C(3)G^Cdel^) abolished the ability for C(3)G to form normal SC along chromosomes, but instead the protein accumulated into aggregates called polycomplexes (PCs). The PCs formed by the C(3)G^Cdel^ protein often take on a hollow cylindrical shape, and may form in either the presence or absence of wild-type C(3)G protein.

**Figure 5:**
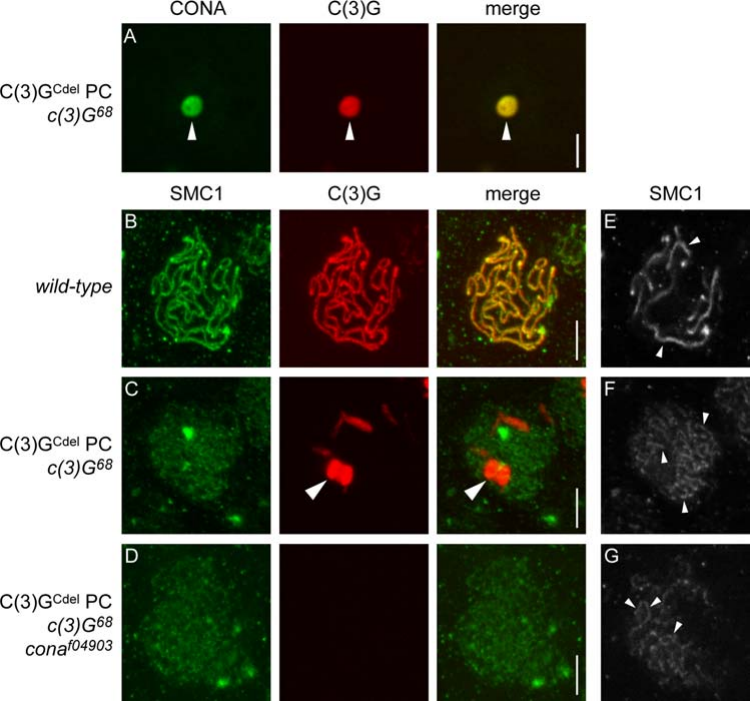
CONA localizes to C(3)G^Cdel^ polycomplexes (PCs) and is required for their formation. (A) A *y w/y w P{nos-GAL4::VP16}*; *P{UASP-c(3)G^Cdel^}4/+*; *c(3)G^68^* pro-oocyte stained to detect CONA (green) and the coiled coil region of C(3)G (red) shows that CONA localization is restricted to the C(3)G^Cdel^ PC (arrowhead). (B) Maximum intensity projections of a wild-type pro-oocyte stained to detect SMC1 (green) and the coiled coil region of C(3)G (red), showing a wild-type pattern of SC. (C) Maximum intensity projections of a *y w/y w P{nos-GAL4::VP16}*; *P{UASP-c(3)G^Cdel^}4/+*; *c(3)G^68^* pro-oocyte stained to detect SMC1 (green) and the coiled coil region of C(3)G (red). Large arrowheads indicate the major C(3)G^Cdel^ PC visible in the nucleus. (D) Maximum intensity projections of a *y w/y w P{nos-GAL4::VP16}*; *P{UASP-c(3)G^Cdel^}4/+*; *c(3)G^68^ cona^f04903^* pro-oocyte stained to detect SMC1 (green) and the coiled coil region of C(3)G (red), demonstrating the lack of PC formation in the absence of CONA. (E) SMC1 localization (white) in a single optical section of the pro-oocyte shown in panel B. (F) SMC1 localization (white) in a single optical section of the pro-oocyte shown in panel C. (G) SMC1 localization (white) in a single optical section of the pro-oocyte shown in panel D. Small arrowheads in E–G indicate thread-like SMC1 localization. Scale bars, 5 µm.

We analyzed CONA localization in C(3)G^Cdel^ PCs by immunofluorescence in females expressing the C(3)G^Cdel^ protein in the absence of wild-type C(3)G. As expected, the C(3)G^Cdel^ protein was detected in sub-cellular bodies of varying size, which correspond to the PCs, and not in a thread-like pattern along chromosomes. Similarly, strong CONA immunofluorescence was detected on the PCs, but not along chromosomes (Figure 5A). This demonstrates that amino acids 651–744 at the C-terminus of C(3)G are dispensable for CONA co-localization and that CONA does not localize along AEs or chromosome cores in the absence of wild-type C(3)G.

Since CONA is necessary for the assembly of wild-type C(3)G into normal SC, and CONA co-localizes with both C(3)G in wild-type and with C(3)G^Cdel^ in PCs, we tested whether CONA is required for the formation of the PCs. Using antibodies specific to the coiled coil region of C(3)G to detect both wild-type C(3)G and C(3)G^Cdel^ (Figure 5B and Figure S2A), we examined germaria from females expressing C(3)G^Cdel^ in a *c(3)G^68^ cona^f04903^* double mutant background. Expression of the C(3)G^Cdel^ protein results in PC formation in a *c(3)G^68^* single mutant background (Figure 5C and Figure S2B). However, when CONA was absent in the *c(3)G^68^ cona^f04903^* double mutant, no anti-C(3)G immunofluorescence was visible above background levels, even though pro-oocyte nuclei could be detected by anti-SMC1 staining (Figure 5D and Figure S2D). The diffuse C(3)G staining observed in *cona* mutants was also not visible in this experiment, possibly due to differences in expression or stability of wild-type C(3)G compared to the C(3)G^Cdel^ protein. As a positive control to ensure that the transgenes encoding GAL4::VP16 and C(3)G^Cdel^ were both present and functioning in the experiment, and that the anti-C(3)G staining was successful, ovaries from sibling *c(3)G^68^ cona^f04903^* heterozygote females were stained and analyzed at the same time. This control, in which both *c(3)G* and *cona* were heterozygous over wild-type alleles, revealed PC formation indicative of C(3)G^Cdel^ expression, as well as thread-like C(3)G staining expected for a *c(3)G cona* double heterozygote (Figure S2C and S2E).

The failure to detect PC formation in *cona* homozygotes demonstrates that CONA is required for C(3)G^Cdel^ PC formation, similar to the requirement of CONA for SC formation. This observation and the localization of CONA to C(3)G^Cdel^ PCs support the hypothesis that CONA is involved in CR formation in SCs. In these experiments we observed the disorganization of chromosomal cores/AEs along chromosome arms when the CR is abrogated by mutations in *c(3)G* and/or *cona*. Chromosomal cores/AEs detected using anti-SMC1 antibodies in wild-type appeared long and thread-like, closely matching C(3)G localization (Figure 5E). In the absence of wild-type C(3)G or CONA, however, SMC1 was detected in less intensely stained linear segments that were more numerous (Figure 5F–G). As noted above, this suggests that assembly of chromosome cores/AEs occurs along the sister chromatids but disruption of the CR of the SC results in disorganization of these structures compared to wild-type.

### Corona Is Necessary for Meiotic Chromosome Pairing

**Figure 6:**
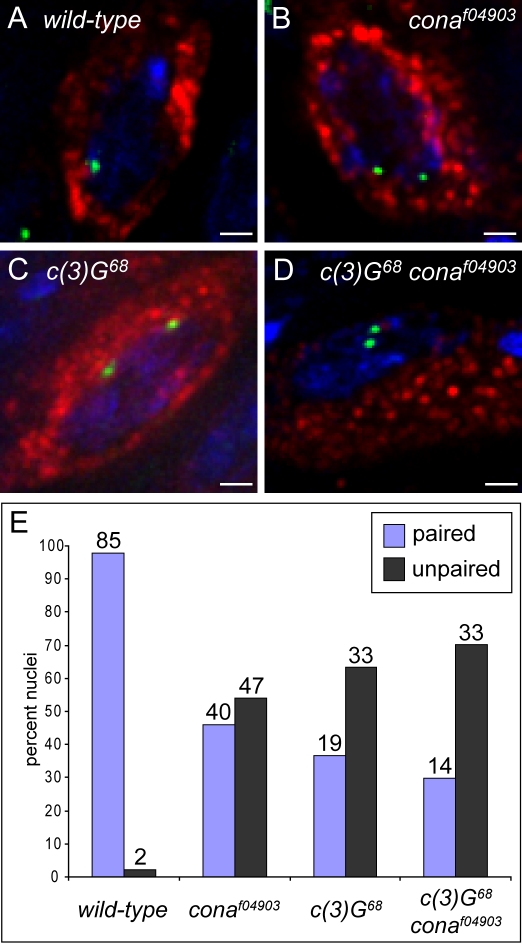
Homologous chromosome pairing is disrupted in *cona* mutants. Shown are pro-oocytes from wild-type (A), *cona^f04903^* (B), *c(3)G^68^* (C), and *c(3)G^68^ cona^f04903^* (D) germaria identified by ORB localization (red) and hybridized with a FISH probe (green) specific for polytene bands 9F4-10B1 of the *X* chromosome. DAPI-stained DNA is shown in blue. In contrast to wild-type (A), in which the FISH signals usually appeared as a single focus or closely spaced foci, FISH signals in *cona^f04903^* were often observed as widely separated foci (B), indicating a disruption in homologous chromosome pairing. Scale bars, 1 µm. (E) Quantified results of the FISH analysis on pro-oocytes and oocytes from germarium regions 2A, 2B, and 3 are shown as percent of nuclei with paired chromosomes (blue bars) and unpaired chromosomes (dark grey bars) in each genotype shown. The number of nuclei observed in each category is shown above each bar. (Nuclei containing a single hybridization focus or foci separated by 0.7 µm or less were defined as paired [33], while those with foci separated by more than 0.7 µm were defined as unpaired.)

The SC is known to play a role in homologous chromosome pairing in Drosophila oocytes [19],[20], and defects in this process could contribute to the disorganization of AEs and the reduction in exchange in *cona* mutants. To determine whether *cona* is required for homologous chromosome pairing, we examined the association of homologous euchromatic DNA sequences in pro-oocytes and oocytes from germarium regions 2A, 2B and 3 using fluorescence *in situ* hybridization (FISH). Using a FISH probe that hybridizes near the middle of the *X* chromosome euchromatin, we found paired homologs in 97.7% (85/87) of the wild-type cells examined (Figure 6A). In contrast, paired homologs were detected in only 46.0% (40/87) of *cona^f04903^* homozygous pro-oocytes and oocytes (Figure 6B). This demonstrates a dramatic decrease in the ability of homologous chromosomes to associate in the absence of CONA.

Testing for homolog pairing in females homozygous for *c(3)G^68^* demonstrated that homologs were paired in only 36.5% (19/52) of cells examined (Figure 6C), which is consistent with previously published results that show a role for C(3)G, and thus the SC, in homolog pairing [19],[20]. In *c(3)G^68^ cona^f04903^* double mutant females, homologs were paired in 29.8% (14/47) of the pro-oocytes and oocytes examined (Figure 6D), a figure not significantly different than that for *c(3)G^68^* alone (χ^2^ = 0.506; p = 0.477). Since CONA is required for normal C(3)G localization, the pairing defect in the *cona* mutant may be a result of abnormal C(3)G localization. We noticed that there was a slight, but not significant (χ^2^ = 3.324; p = 0.068), elevation in pairing frequency in *cona^f04903^* homozygotes compared to *c(3)G^68^ cona^f04903^* double homozygotes, which could possibly be explained by a low level of homolog pairing promoted by the small amount of C(3)G that localizes to chromosomes in the *cona^f04903^* single mutant (Figure 4). These data demonstrate that both *c(3)G* and *cona* are necessary for normal levels of homolog pairing, and are consistent with CONA functioning within the CR of the SC to promote synapsis.

## Discussion

### Corona Is Critical for Polymerization of C(3)G to Form the Central Region of the SC

We have characterized Corona (CONA), a novel SC-associated protein that is critical for the higher-order assembly of TFs into the CR of the SC. The normal localization of CONA and C(3)G is mutually-dependent – in the absence of CONA, C(3)G is visible as only spots or short threads along meiotic chromosome cores, and in the absence of C(3)G, CONA appears to be absent from the meiotic nucleus. Three lines of evidence suggest that CONA plays a critical role in the stable assembly of C(3)G into the CR of the SC. First, *cona* mutant oocytes fail to form long stretches of continuous SC, and only short threads or spots of C(3)G are visible within the pro-oocyte nucleus (Figure 4 and Figure S1). Second, the dependence of SC assembly (as assayed by C(3)G polymerization) on CONA::Venus expression in the absence of endogenous CONA, as well as the co-localization of CONA and C(3)G in *c(2)M* and *ord* mutants (Figure 2 and Figure 3) suggest that CONA is required to polymerize C(3)G into long stretches required to form the CR. Third, the requirement for CONA to facilitate C(3)G polymerization is also demonstrated by the fact that CONA localizes to the C(3)G PCs created by expressing C(3)G proteins that lack their C-termini and thus cannot bind chromosomes (Figure 5). Moreover, CONA also is required for the formation of these PCs, demonstrating that CONA has a functional role necessary for the connection of C(3)G^Cdel^ molecules in PCs.

The phenotypes of *cona* mutants make it clear that the CONA-mediated assembly of C(3)G into polymerized TFs is required for most, if not all, aspects of C(3)G function. Despite being present in *cona* mutants, C(3)G protein is unable to promote homolog synapsis or exchange. Defects in meiotic pairing, synapsis, and recombination are similar in *cona*, *c(3)G* and *c(3)G cona* mutant pro-oocytes (Figure 6, SLP and RSH, unpublished data).

### How Might CONA Function?

In terms of its role in the formation of the CR of the SC, CONA may have a role similar to the mouse CE proteins SYCE1, SYCE2, and TEX12 [23]–[26]. These proteins co-localize extensively with the TF protein SYCP1, though SYCE2 and TEX12 were reported to have a more punctate appearance. Moreover, SYCE1 and SYCE2 also remain co-localized with SYCP1 when AEs/LEs are disrupted in *Sycp3* ^−/−^ spermatocytes and oocytes, and are unable to localize to meiotic chromosomes in the absence of SYCP1 [24],[25]. Mutation of SYCE2 or TEX12 disrupts synapsis and greatly reduces the amount of SYCP1 that localizes to chromosomes, yet AE proteins localize normally. In *Syce2^−/−^* and *Tex12^−/−^* meiotic cells, synapsis appears to be initiated at multiple sites along the paired homologs, but they fail to extend and form full-length SC [23],[26]. These findings are quite similar to the *cona* mutant phenotype, in which only a small amount of C(3)G is found on chromosomes, while the C(2)M, SMC1, and ORD proteins are still localized properly.

SYCE1 has been proposed to stabilize head-to-head interactions between SYCP1 dimers, while SYCE2 and TEX12 have been proposed to connect SYCP1-SYCE1 complexes to form higher-order structures [23],[26]. Either of these roles of CE proteins is consistent with the activities of CONA, in that the N-terminus of C(3)G is localized to the CE and required for normal SC formation [12],[21] and the formation of higher order SC or PC structures fails in the absence of CONA. Moreover, the phenotype exhibited by *cona* mutants parallels that documented for N-terminal deletions of C(3)G [21]; only spots or short stretches of chromosomally-associated C(3)G are visible. These data suggest that either one large or multiple small domains deleted in these N-terminus-deficient C(3)G proteins may define regions of C(3)G that interact with CONA.

### *cona* and *c(3)G* Mutations Both Abolish Alignment of the AEs

Localization of C(2)M, SMC1, and ORD in *cona* mutant pro-oocytes indicates that chromosome core/AE structures are present, although they are more numerous and appear thinner than in wild-type. This disorganized pattern resembles that observed for C(2)M and cohesin SMC proteins when C(3)G is absent [38],[39] and argues that AEs cannot align in the absence of synapsis in Drosophila oocytes. In addition, FISH analysis demonstrates that pairing of homologous sequences is severely disrupted in *cona* (this study) and *c(3)G* oocytes [19],[20].

Disruption of homolog pairing and alignment in *cona* and *c(3)G* mutants contrasts sharply with what is observed in mammalian meiocytes lacking the TF protein SYCP1 or CE proteins SYCE2 or TEX12. Although homologous chromosomes fail to synapse in *Sycp1^−/−^*, *Syce2^−/−^*, and *Tex12^−/−^* meiotic cells, AEs lie in close proximity along their entire length [18],[23],[26]. Presumably, the formation of DSBs at multiple sites along the chromosomes establishes axial associations and these are sufficient to hold homologous chromosomes in close proximity even when the SC fails to propagate [18],[23],[26]. Axial associations likely form the basis for the assembly of the short regions of SC observed in *Syce2^−/−^* and *Tex12^−/−^* meiotic cells, which could further secure the alignment of homologs. While we cannot rule out the possibility that similar short regions of “synapsis” exist in *cona* oocytes, it seems likely that even a small number of these along the length of the chromosome would result in at least some examples in which AEs lie as “parallel tracks” in chromosome spreads, a phenomenon that we did not observe.

Our analysis of *cona* mutant oocytes suggests that, unlike mammals, the SC is critical for early events governing the pairing/alignment of homologous chromosomes in Drosophila. We can envision at least three different models that might explain why homolog alignment is dependent on SC in Drosophila. In the first model, homologous chromosomes enter meiotic prophase already paired and aligned as a result of the persistence of pairing established during preceding cell cycles and the rapid formation of SC is required to maintain these associations [4]. Although this model has been favored in the past, two published reports refute the argument that homologous chromosomes enter meiosis already paired and aligned. As noted by Fung and colleagues [45] as well as Csink and Henikoff [46], the pairing of homologous chromosomes in Drosophila somatic cells is disrupted during both replication and mitosis. Therefore, any pairing that exists prior to meiotic S phase would be lost and need to be re-established, most likely during meiotic prophase.

The second model posits that the different effects on homolog pairing and alignment observed in flies and mammals reflect differences in the ability of CE proteins to stabilize short stretches of SC. In contrast to flies, DSBs are required for synapsis in mice [47]–[49]. The short stretches of SC resulting from the formation of DSBs and early recombination intermediates in mouse meiocytes lacking SYCE2 or TEX12 may maintain the alignment of AEs in the absence of full synapsis. If the requirement for CE function is sufficiently more stringent in flies than in mammals, then short regions of synapsis similar to those observed in *Tex12^−/−^* and *Syce2^−/−^* meiocytes may be unstable or never form in *cona* mutant flies. In the absence of such stretches of SC or DSB-induced axial associations, the Drosophila homologs would be expected to quickly dissociate.

Our third model is based on the different temporal relationship between DSB formation and SC assembly in flies and mammals. In mammals, DSB formation and the formation of early recombination intermediates occur commensurate with, and are required for SC formation [47],[48]. In contrast, DSB initiation occurs after the completion of SC assembly in Drosophila and is not required for synapsis [49]–[51]. Because SC assembly in flies occurs via a DSB-independent pathway, pairing/alignment of AEs may be abolished in mutant oocytes in which higher-order assembly of TFs is prevented. According to this model, initial pre-synaptic associations of homologs may be maintained either by the formation of early recombination intermediates and axial associations that lead to the initiation of short stretches of SC (the mammalian paradigm), or by the establishment of extensive synapsis (the Drosophila paradigm). In both cases, the initial event (formation of recombination intermediates or SC formation) is eventually followed and perhaps ‘locked-in’ by the other. One could hypothesize that mammalian CE mutants can maintain alignments because of the earlier formation of recombination intermediates and axial associations. In contrast, lack of SC assembly in *cona* and *c(3)G* mutants would compromise the essential early step that maintains the alignment of homologous chromosomes in Drosophila oocytes. If the homologs are already apart by the time DSBs occur in *cona* and *c(3)G* mutants, DSBs would be too late to stabilize homolog associations and maintain AE alignment.

In summary, our data demonstrate an essential requirement for CONA in the polymerization of C(3)G that is required for SC formation. Understanding the mechanism by which CONA performs that role will require the identification of CONA-interacting proteins, which we expect will include the N-terminal globular domain of C(3)G and perhaps other CE proteins as well. Elucidating the function of these proteins in SC assembly and the consequences of their loss by mutation may also help us understand the role of the SC in establishing or maintaining the pairing and alignment of homologs in early prophase.

## Materials and Methods

### Drosophila Stocks and Genetic Analyses

Drosophila stocks and crosses were maintained on a standard medium at 25°C. Descriptions of genetic markers and chromosomes can be found at http://www.flybase.org/ [52]. A *w^1118^* stock was used as a wild-type stock for the immunofluorescence and FISH experiments, except for the experiment shown in Figure 1A, in which a *Canton-S* strain was used. *Df(3R)JDP* was constructed by FLP-mediated recombination essentially as described by Parks *et al.* [53] using FRT sequences in *PBac{WH}cona^f04903^* and *P{XP}d01968*, inserted at coordinates 14,211,754 and 14,222,824, respectively, on the chromosome *3R* genome map (Release 5.6). The entire *cona* protein-coding region is deleted in *Df(3R)JDP*.

The transgene construct *P{UASP-cona::Venus}* was constructed using the plasmid pPWV (obtained from the Drosophila Genomics Resource Center, Bloomington, IN) and the Gateway system (Invitrogen, Carlsbad, CA) using methods as recommended by the manufacturer. pPWV is identical to pUASP except that it contains a Gateway cassette followed by the Venus yellow fluorescent protein coding region [54]. The *cona* open reading frame was amplified from the *cona* cDNA bs15d10 (obtained from Geneservice, Ltd., Cambridge, UK) using primers tailed with *att*B1 and *att*B2 sequences and inserted into the vector pDONR221 in a BP Clonase (Invitrogen) reaction to form pDONR-*cona*. The *cona* cDNA insert from pDONR-*cona* was then transferred into pPWV in an LR Clonase (Invitrogen) reaction to form pP{UASP-*cona::Venus*}, with an open reading frame encoding a CONA::Venus fusion protein. After confirming the construct by sequence analysis, it was introduced into Drosophila by standard germline transformation methods (Genetic Services, Inc., Cambridge, MA).

To observe GFP-ORD in chromosome spread experiments, *P{gc(2)M-myc}II.5 P{GFP::ORD}48I ord^10^ bw sp If/+*; *cona^f04903^ e^s^ ca /FRT82B cona^A12^* females were obtained by crossing *y w/y^+^Y; (P{gc(2)M-myc}II.5 P{GFP::ORD}48I ord^10^ bw sp If*; *cona^f04903^ e^s^ ca)/T(2;3)CyO-TM3*, *P{GAL4-Hsp70.PB}TR1*, *P{UAS-GFP.Y}TR1: P{GAL4-Hsp70.PB}TR2*, *P{UAS-GFP.Y}TR2*, *Ser^1^* males to *y^d2^ w^1118^ P{ey-FLP.N}2 P{GMR-lacZ.C(38.1)}TPN1/Y*; *FRT82B cona^A12^/TM6B*, *P{y^+^}TPN1*, *Tb^1^* females. For chromosome spread experiments to observe C(2)M, homozygous *cona^A12^* females were selected from the stock *y^d2^ w^1118^ P{ey-FLP.N}2 P{GMR-lacZ.C(38.1)}TPN1/Y*; *FRT82B cona^A12^/TM6B, P{y^+^}TPN1*, *Tb^1^*.

### Antibody Production

The full-length *cona* open reading frame was amplified from the *cona* cDNA bs15d10 and cloned into pET-19b (Novagen, San Diego, CA). After the construct was verified by sequencing, the 6XHis-tagged CONA protein was expressed in *E. coli* BL21 cells. The bacterial expressed protein was purified using ProBond Nickel-Chelating Resin (Invitrogen). Polyclonal antibody production in guinea pigs using purified 6XHis-CONA as antigen was performed by Cocalico Biologicals (Reamstown, PA). Pre-immune sera from the immunized guinea pigs did not stain Drosophila ovaries (data not shown).

The anti-CONA antibody was specific to the CONA protein, as anti-CONA signals were not detected in ovaries from *cona^f04903^* females (Figure S1B). Similar observations were made using ovaries from *cona^A12^*/*Df(3R)JDP* females [SLP and WDW, unpublished data]. These observations suggested that little or no endogenous CONA protein is produced in the presence of the *cona^A12^* or *cona^f04903^* mutations.

### Immunofluorescence on Whole-Mount Ovarioles

Immunofluorescence on whole ovarioles was performed as described previously and the ovarioles were mounted on coverslips by embedding in polyacrylamide gel in most experiments [8]. Primary antibodies used for staining whole-mount preparations were guinea pig anti-CONA (1∶125), mouse monoclonal anti-C(3)G 1A8-1G2 [12] (1∶500), mouse monoclonal anti-C(3)G 1G5-2F7 and 5G4-1F1 [12],[21] (1∶500 each), mouse monoclonal anti-ORB 6H4 and 4H8 [55] (1∶50 each), and rat anti-SMC1 [56] (1∶500). Secondary antibodies were Alexa 546 anti-mouse IgG (1∶500), Alexa 488 anti-mouse IgG (1∶500), Alexa 488 anti-guinea pig IgG (1∶500), Alexa 488 anti-rat IgG (1∶500) (Invitrogen), and Cy3 anti-mouse IgG (1∶500) (Jackson Immunoresearch, West Grove, PA).

Microscopy was conducted using a DeltaVision RT restoration microscopy system (Applied Precision, Issaquah, WA) equipped with an Olympus IX70 inverted microscope and CoolSnap CCD camera. Image data were corrected and deconvolved using softWoRx v.2.5 software (Applied Precision). For some experiments, confocal images were collected using a Bio-Rad Radiance 2000 laser scanning confocal microscope and Zeiss LaserSharp2000 software. Maximum intensity projections were produced from confocal data using Zeiss LSM Image Browser v.4.2 software.

### Immunofluorescence on Chromosome Spreads

Chromosome spread experiments were performed as described previously [38]. Primary antibodies used for immunofluorescence on chromosome spreads were affinity-purified guinea pig anti-SMC1 [38] (1∶500), rabbit anti-C(2)M [39] (1∶500), rabbit anti-GFP (Invitrogen) (1∶500), and mouse monoclonal anti-C(3)G 1A8-1G2 [12] (1∶500). Secondary antibodies were Alexa 488 anti-rabbit IgG (1∶400), Alexa 488 anti-mouse IgG (1∶400) (Invitrogen), Cy3 anti-guinea pig IgG (1∶400), Cy5 anti-guinea pig IgG (1∶400), and Cy5 anti-mouse IgG (1∶400) (Jackson Immunoresearch).

For chromosome spreads, images were captured and processed as described previously [38]. Because the signal intensity varies considerably for different nuclei on the same slide, wild-type and mutant images were enhanced to different degrees during processing to render details visible. In general, the C(3)G signal on chromatin in *cona* nuclei is significantly weaker than in wild-type.

### Fluorescence In Situ Hybridization (FISH)

FISH on ovarioles was performed as described elsewhere [57] with simultaneous immunofluorescence detection of ORB protein. The probe for the FISH experiments was composed of three overlapping bacterial artificial chromosome (BAC) clones from the RP98 library [58] obtained from the BACPAC Resource Center, Children's Hospital Oakland Research Institute. The three BACs (and map locations on the *X* chromosome) were RP98-26N1 (9F4-10A2), RP98-17B23 (9F11-10A4), and RP98-26J12 (10A4-B1). BAC DNA was isolated using the Qiagen Midi Prep Kit. A DNA mixture containing 3.3 µg of DNA from each of the three BACs was labeled with Alexa 488 (Invitrogen) essentially as described by Dernburg [59] and purified using a Qiaquick column (Qiagen). Immunofluorescence with anti-ORB primary antibodies and Cy3 anti-mouse IgG secondary antibodies was performed after hybridization under the same conditions as described above for whole mount ovarioles. The ovarioles were mounted in Prolong Gold antifade mountant (Invitrogen) [60].

Images were collected using a DeltaVision RT restoration microscopy system as described above. After image collection and processing, hybridization foci within pro-oocyte nuclei were scored for chromosome pairing. In nuclei with two foci, the distance between the pixels of highest fluorescence intensity within each focus was measured in three-dimensional image stacks using softWoRx Explorer software (Applied Precision). Nuclei containing a single hybridization focus or foci separated by 0.7 µm or less were defined as paired [19], while those with foci separated by more than 0.7 µm were defined as unpaired.

## Supporting Information

Figure S1 CONA and C(3)G localization in *cona* mutant pro-oocytes. (A) Wild-type control pro-oocytes showing CONA and C(3)G co-localization. (B) *cona^f04903^* homozygous pro-oocytes showing CONA is not detected and C(3)G localization is more diffuse than in wild-type nuclei with threads that are less distinct. Similar observations were made using ovaries from *cona^A12^*/*Df(3R)JDP* females (SLP and WDW, unpublished data). These observations indicate that little or no endogenous CONA protein is produced in the presence of the *cona^A12^* or *cona^f04903^* mutations. (C) *cona^f04903^/+* pro-oocytes in early region 2A showing CONA is present and co-localizes with the polymerizing C(3)G in early zygotene stage pro-oocytes (arrow) that show spotty C(3)G localization. (D) *cona^f04903^/+* pro-oocytes in late region 2A showing that CONA is present and co-localized with C(3)G, similar to wild-type. Pro-oocytes were stained with anti-CONA (green) and anti-C(3)G (red). Each image represents a single deconvolved optical section. Scale bars, 2.5 µm (A, C, D) and 5 µm (B).
(5.2 MB TIF)

Figure S2 CONA is required for C(3)G^Cdel^ polycomplex (PC) formation. (A) Maximum intensity projections of a wild-type germarium stained to detect SMC1 (green) and the coiled coil region of C(3)G (red). Arrowheads indicate pro-oocytes with thread-like C(3)G localization. (B) Maximum intensity projections of a *y w/y w P{nos-GAL4::VP16}*; *P{UASP-c(3)G^Cdel^}4/+*; *c(3)G^68^* germarium stained to detect SMC1 (green) and the coiled coil region of C(3)G (red). Arrowheads indicate PCs visible in pro-oocyte nuclei. (C) Maximum intensity projections of a *y w/y w P{nos-GAL4::VP16}*; *P{UASP-c(3)G^Cdel^}4/+*; *c(3)G^68^ cona^f04903^/TM3*, *Ser* germarium stained to detect SMC1 (green) and the coiled coil region of C(3)G (red). Arrowheads indicate PCs visible in pro-oocyte nuclei that also have thread-like C(3)G localization due to heterozygosity for *c(3)G^68^* and *cona^f04903^*. (D) Maximum intensity projections of a *y w/y w P{nos-GAL4::VP16}*; *P{UASP-c(3)G^Cdel^}4/+*; *c(3)G^68^ cona^f04903^* germarium stained to detect SMC1 (green) and the coiled coil region of C(3)G (red), which demonstrates the lack of PC formation in the absence of CONA. (E) Maximum intensity projections of a *y w/y w P{nos-GAL4::VP16}*; *P{UASP-c(3)G^Cdel^}4/+*; *c(3)G^68^ cona^f04903^/TM3*, *Ser* pro-oocyte stained to detect SMC1 (green) and the coiled coil region of C(3)G (red). Large arrowheads indicate the major PC visible in the nucleus. Small arrowheads indicate thread-like C(3)G localization also present due to heterozygosity for *c(3)G^68^* and *cona^f04903^*. Scale bars, 50 µm (A-D), 5 µm (E).
(3.9 MB TIF)
