# Supplementary figures and images for: corona Is Required for Higher-Order Assembly of Transverse Filaments into Full-Length Synaptonemal Complex in Drosophila Oocytes
Source: PLoS Genet. 2008 Sep 19;4(9):e1000194. doi: 10.1371/journal.pgen.1000194 (PMC2529403; doi:10.1371/journal.pgen.1000194)

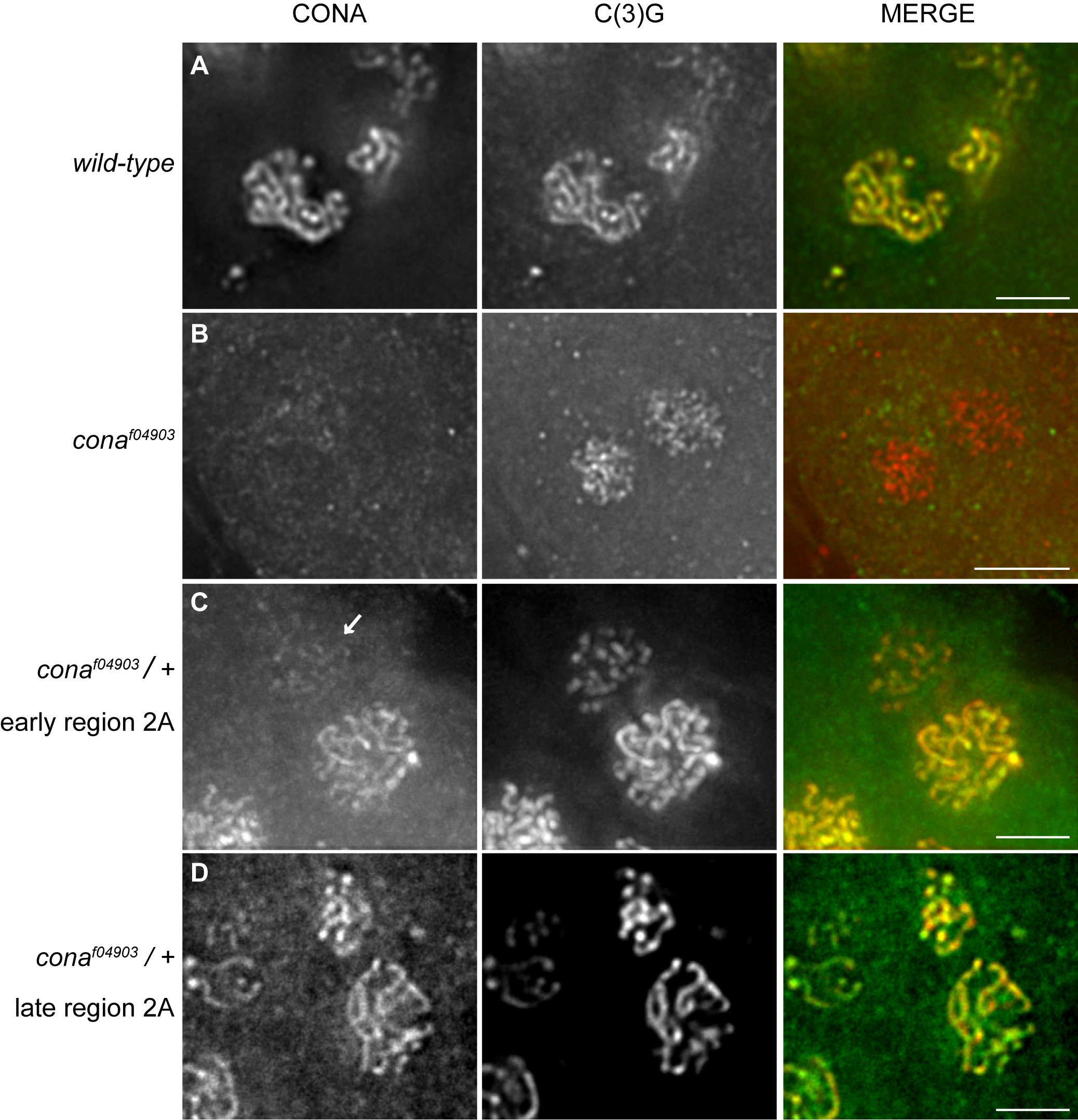

Supplement: Figure S1 — CONA and C(3)G localization in cona mutant pro-oocytes. (A) Wild-type control pro-oocytes showing CONA and C(3)G co-localization. (B) conaf04903 homozygous pro-oocytes showing CONA is not detected and C(3)G localization is more diffuse than in wild-type nuclei with threads that are less distinct. Similar observations were made using ovaries from conaA12/Df(3R)JDP females (SLP and WDW, unpublished data). These observations indicate that little or no endogenous CONA protein is produced in the presence of the conaA12 or conaf04903 mutations. (C) conaf04903/+ pro-oocytes in early region 2A showing CONA is present and co-localizes with the polymerizing C(3)G in early zygotene stage pro-oocytes (arrow) that show spotty C(3)G localization. (D) conaf04903/+ pro-oocytes in late region 2A showing that CONA is present and co-localized with C(3)G, similar to wild-type. Pro-oocytes were stained with anti-CONA (green) and anti-C(3)G (red). Each image represents a single deconvolved optical section. Scale bars, 2.5 µm (A, C, D) and 5 µm (B). (5.2 MB TIF) [file pgen.1000194.s001.tif]

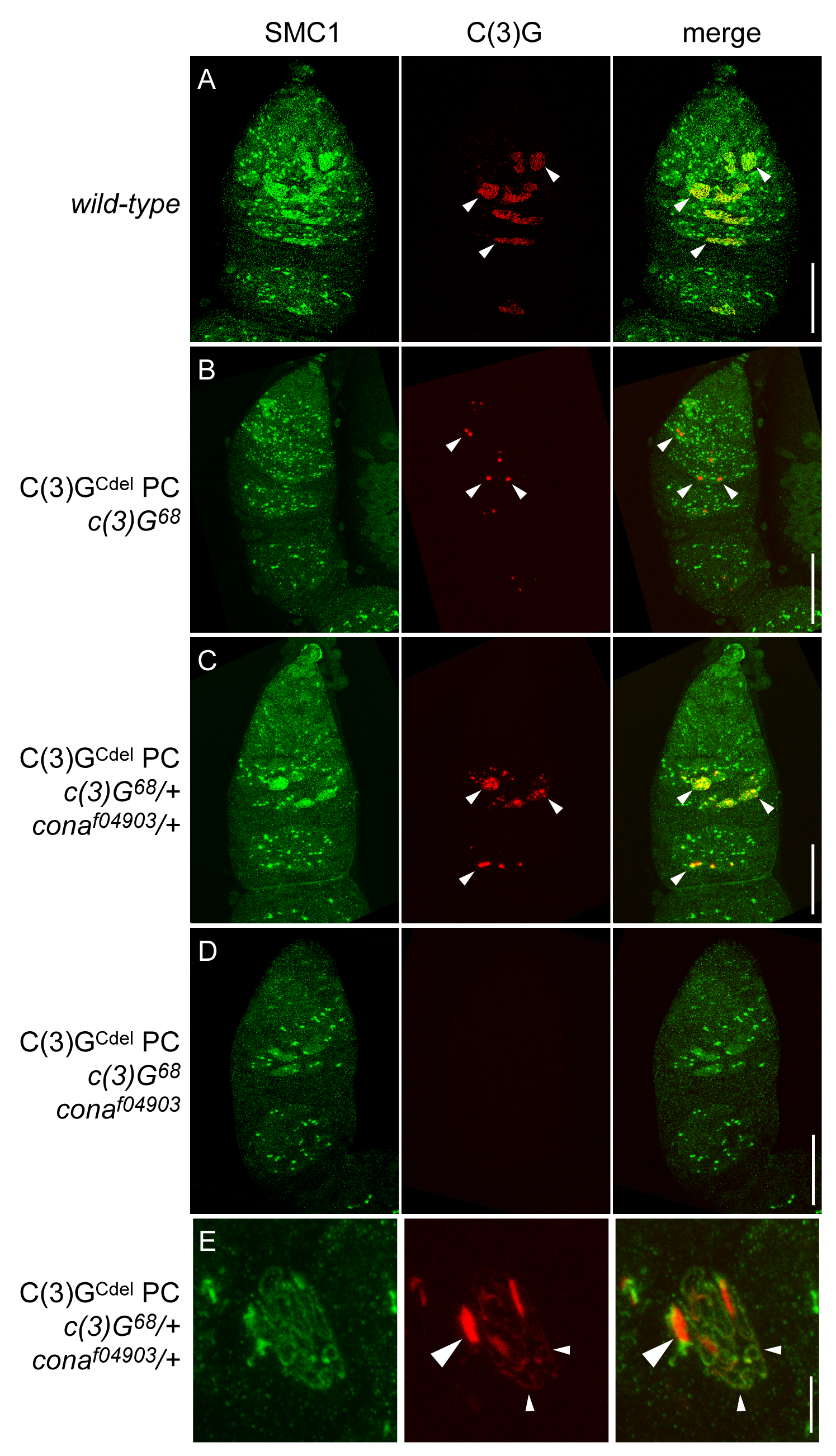

Supplement: Figure S2 — CONA is required for C(3)GCdel polycomplex (PC) formation. (A) Maximum intensity projections of a wild-type germarium stained to detect SMC1 (green) and the coiled coil region of C(3)G (red). Arrowheads indicate pro-oocytes with thread-like C(3)G localization. (B) Maximum intensity projections of a y w/y w P{nos-GAL4::VP16}; P{UASP-c(3)GCdel}4/+; c(3)G68 germarium stained to detect SMC1 (green) and the coiled coil region of C(3)G (red). Arrowheads indicate PCs visible in pro-oocyte nuclei. (C) Maximum intensity projections of a y w/y w P{nos-GAL4::VP16}; P{UASP-c(3)GCdel}4/+; c(3)G68 conaf04903/TM3, Ser germarium stained to detect SMC1 (green) and the coiled coil region of C(3)G (red). Arrowheads indicate PCs visible in pro-oocyte nuclei that also have thread-like C(3)G localization due to heterozygosity for c(3)G68 and conaf04903. (D) Maximum intensity projections of a y w/y w P{nos-GAL4::VP16}; P{UASP-c(3)GCdel}4/+; c(3)G68 conaf04903 germarium stained to detect SMC1 (green) and the coiled coil region of C(3)G (red), which demonstrates the lack of PC formation in the absence of CONA. (E) Maximum intensity projections of a y w/y w P{nos-GAL4::VP16}; P{UASP-c(3)GCdel}4/+; c(3)G68 conaf04903/TM3, Ser pro-oocyte stained to detect SMC1 (green) and the coiled coil region of C(3)G (red). Large arrowheads indicate the major PC visible in the nucleus. Small arrowheads indicate thread-like C(3)G localization also present due to heterozygosity for c(3)G68 and conaf04903. Scale bars, 50 µm (A-D), 5 µm (E). (3.9 MB TIF) [file pgen.1000194.s002.tif]
